# 2,5-Dihy­droxy­terephthalic acid dihydrate

**DOI:** 10.1107/S1600536810025766

**Published:** 2010-07-07

**Authors:** Po-Wen Cheng, Chi-Feng Cheng, Yeh Chun-Ting, Chia-Her Lin

**Affiliations:** a Department of Chemistry, Chung-Yuan Christian University, Chung-Li 320, Taiwan

## Abstract

The title compound, C_8_H_6_O_6_·2H_2_O, was obtained by accident within a project on the synthesis of metal–organic coordination polymers by the reaction of LiOH with 2,5-dihy­droxy­terephthalic acid under solvothermal conditions. The asymmetric unit consists of half a 2,5-dihy­droxy­terephthalic acid mol­ecule located on a centre of inversion and one solvent water mol­ecule that occupies a general position. The 2,5-dihy­droxy­terephthalic acid mol­ecules are connected to the water mol­ecules *via* O—H⋯O hydrogen bonding to form a layer in the *ab* plane.

## Related literature

For genernal background to supramolecular assembly and crystal engineering, see: Kitagawa *et al.* (2004 ▶).
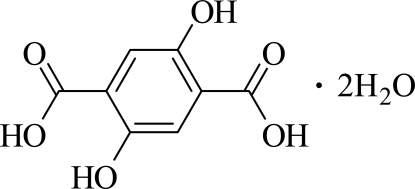


## Experimental

### 

#### Crystal data

C_8_H_6_O_6_·2H_2_O
*M* *_r_* = 234.16
Monoclinic, 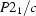

*a* = 5.1883 (10) Å
*b* = 17.545 (4) Å
*c* = 5.4990 (12) Å
β = 103.03 (1)°
*V* = 487.68 (17) Å^3^
*Z* = 2
Mo *K*α radiation
μ = 0.15 mm^−1^
*T* = 295 K
0.25 × 0.20 × 0.20 mm

#### Data collection

Bruker APEXII CCD diffractometer
Absorption correction: multi-scan (*SADABS*; Bruker, 2009 ▶) *T*_min_ = 0.945, *T*_max_ = 0.963
4475 measured reflections
1208 independent reflections
589 reflections with *I* > 2σ(*I*)
*R*_int_ = 0.080

#### Refinement

*R*[*F* ^2^ > 2σ(*F* ^2^)] = 0.061
*wR*(*F* ^2^) = 0.193
*S* = 1.02
1208 reflections
73 parameters
H-atom parameters constrained
Δρ_max_ = 0.36 e Å^−3^
Δρ_min_ = −0.32 e Å^−3^

### 

Data collection: *APEX2* (Bruker, 2009 ▶); cell refinement: *SAINT* (Bruker, 2009 ▶); data reduction: *SAINT*; program(s) used to solve structure: *SHELXS97* (Sheldrick, 2008 ▶); program(s) used to refine structure: *SHELXL97* (Sheldrick, 2008 ▶); molecular graphics: *SHELXTL* (Sheldrick, 2008 ▶); software used to prepare material for publication: *SHELXTL*.

## Supplementary Material

Crystal structure: contains datablocks I, global. DOI: 10.1107/S1600536810025766/nc2191sup1.cif

Structure factors: contains datablocks I. DOI: 10.1107/S1600536810025766/nc2191Isup2.hkl

Additional supplementary materials:  crystallographic information; 3D view; checkCIF report

## Figures and Tables

**Table 1:** Hydrogen-bond geometry (Å, °)

*D*—H⋯*A*	*D*—H	H⋯*A*	*D*⋯*A*	*D*—H⋯*A*
O1—H1*A*⋯O3^i^	0.82	1.88	2.597 (3)	146
O2—H2*B*⋯O1*W*^ii^	0.82	1.74	2.561 (3)	177
O1*W*—H1*WB*⋯O1^iii^	0.85	1.94	2.786 (3)	175.0
O1*W*—H1*WA*⋯O3^iv^	0.85	2.04	2.809 (3)	150.4

Symmetry codes: (i) 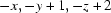; (ii) 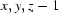; (iii) 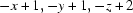; (iv) 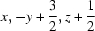.

**Fig. 1.**
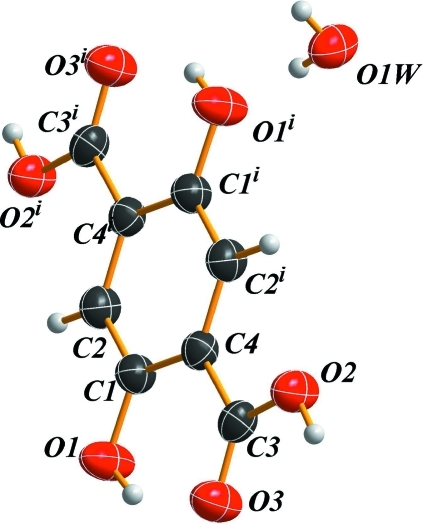
The molecular structure view of the title compound with labelling and displacement ellipsoids drawn at the 50% probability level. [symmetry codes: (i) -x, 1 - y, 2 - z].

**Fig. 2.**
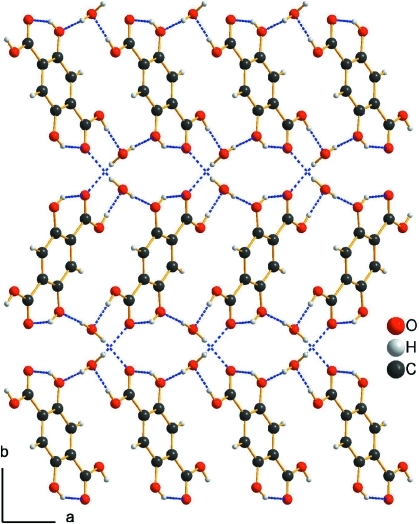
Crystal structure of title compound with view along a-axis. Hydrogen bonding is shown as blue dashed lines.

## supplementary crystallographic information

### Experimental

The solvothermal reactions were carried out in Teflon-lined digestion bombs (internal volume of 23 ml) under autogenously pressure by heating the reaction mixtures followed by slow cooling at 6 K h^-1^ to room temperature. Crystals of the title compound were obtained from the reaction of 2,5-dihydroxyterephthalic acid (C_8_H_4_O_6_, 0.198 g, 1.0 mmol) with Li(OH) (0.048 g, 2.0 mmol) in H_2_O (10.0 ml). The mixture was heated at 363 K for 3 d. On cooling light-yellow crystals had formed.

### Refinement

The H atoms of the benzene rings were placed in idealized positions and constrained to ride on their parent atoms, with C—H = 0.93 Å and *U*_iso_(H) = 1.2*U*_eq_(C). The hydroxyl H atoms of the carboxyl groups were placed in ideal positions with the O—H bond trans to the longest bond of the adjacent atom (O—H = 0.82 Å) and refined using a riding model. One H atom of the water molecule were located in difference map, the other placed in an ideal position in order that reasonable hydrogen bonding is found. Finally they were refined using a riding model with O—H = 0.85 Å. All O—H H atoms were refined with *U*_iso_(H) = 1.2*U*_eq_(O).

### Crystal data

**Table:**

C_8_H_6_O_6_·2H_2_O	*F*(000) = 244
*M**_r_* = 234.16	*D*_x_ = 1.595 Mg m^−^^3^
Monoclinic, *P*2_1_/*c*	Mo *K*α radiation, λ = 0.71073 Å
*a* = 5.1883 (10) Å	Cell parameters from 760 reflections
*b* = 17.545 (4) Å	θ = 2.3–22.5°
*c* = 5.4990 (12) Å	µ = 0.15 mm^−^^1^
β = 103.03 (1)°	*T* = 295 K
*V* = 487.68 (17) Å^3^	Tablular, light-yellow
*Z* = 2	0.25 × 0.20 × 0.20 mm

### Data collection

**Table:**

Bruker APEXII CCD diffractometer	1208 independent reflections
Radiation source: fine-focus sealed tube	589 reflections with *I* > 2σ(*I*)
graphite	*R*_int_ = 0.080
Detector resolution: 8.3333 pixels mm^-1^	θ_max_ = 28.4°, θ_min_ = 2.3°
φ and ω scans	*h* = −5→6
Absorption correction: multi-scan (*SADABS*; Bruker, 2009)	*k* = −23→23
*T*_min_ = 0.945, *T*_max_ = 0.963	*l* = −7→4
4475 measured reflections	

### Refinement

**Table:**

Refinement on *F*^2^	Primary atom site location: structure-invariant direct methods
Least-squares matrix: full	Secondary atom site location: difference Fourier map
*R*[*F*^2^ > 2σ(*F*^2^)] = 0.061	Hydrogen site location: inferred from neighbouring sites
*wR*(*F*^2^) = 0.193	H-atom parameters constrained
*S* = 1.02	*w* = 1/[σ^2^(*F*_o_^2^) + (0.0851*P*)^2^] where *P* = (*F*_o_^2^ + 2*F*_c_^2^)/3
1208 reflections	(Δ/σ)_max_ = 0.009
73 parameters	Δρ_max_ = 0.36 e Å^−^^3^
0 restraints	Δρ_min_ = −0.32 e Å^−^^3^

### Special details

**Table:**

Geometry. All e.s.d.'s (except the e.s.d. in the dihedral angle between two l.s. planes) are estimated using the full covariance matrix. The cell e.s.d.'s are taken into account individually in the estimation of e.s.d.'s in distances, angles and torsion angles; correlations between e.s.d.'s in cell parameters are only used when they are defined by crystal symmetry. An approximate (isotropic) treatment of cell e.s.d.'s is used for estimating e.s.d.'s involving l.s. planes.
Refinement. Refinement of *F*^2^ against ALL reflections. The weighted *R*-factor *wR* and goodness of fit *S* are based on *F*^2^, conventional *R*-factors *R* are based on *F*, with *F* set to zero for negative *F*^2^. The threshold expression of *F*^2^ > σ(*F*^2^) is used only for calculating *R*-factors(gt) *etc*. and is not relevant to the choice of reflections for refinement. *R*-factors based on *F*^2^ are statistically about twice as large as those based on *F*, and *R*- factors based on ALL data will be even larger.

### Fractional atomic coordinates and isotropic or equivalent isotropic displacement parameters (Å^2^)

**Table:**

	*x*	*y*	*z*	*U*_iso_*/*U*_eq_	
O1	0.0253 (5)	0.35101 (13)	0.8449 (4)	0.0561 (7)	
H1A	−0.0615	0.3231	0.9153	0.084*	
O2	0.4188 (5)	0.59034 (13)	0.6391 (4)	0.0488 (7)	
H2B	0.4915	0.6261	0.5857	0.073*	
O3	0.2796 (5)	0.68281 (14)	0.8554 (4)	0.0536 (7)	
O1W	0.6537 (4)	0.69917 (13)	1.4663 (4)	0.0524 (7)	
H1WA	0.5399	0.7288	1.3796	0.079*	
H1WB	0.7486	0.6811	1.3724	0.079*	
C1	0.0087 (6)	0.42428 (18)	0.9234 (5)	0.0371 (8)	
C2	0.1432 (6)	0.48027 (18)	0.8263 (5)	0.0394 (9)	
H2A	0.2394	0.4671	0.7089	0.047*	
C3	0.2843 (6)	0.61572 (19)	0.7961 (5)	0.0378 (8)	
C4	0.1382 (6)	0.55608 (17)	0.9001 (5)	0.0337 (8)	

### Atomic displacement parameters (Å^2^)

**Table:**

	*U*^11^	*U*^22^	*U*^33^	*U*^12^	*U*^13^	*U*^23^
O1	0.0761 (17)	0.0361 (15)	0.0720 (15)	−0.0063 (12)	0.0504 (14)	−0.0070 (12)
O2	0.0610 (15)	0.0415 (15)	0.0545 (14)	−0.0033 (11)	0.0352 (12)	0.0004 (11)
O3	0.0678 (17)	0.0402 (15)	0.0639 (16)	−0.0083 (12)	0.0380 (13)	−0.0066 (12)
O1W	0.0646 (16)	0.0461 (16)	0.0575 (14)	0.0068 (12)	0.0371 (13)	0.0091 (12)
C1	0.0391 (18)	0.037 (2)	0.0386 (16)	0.0007 (14)	0.0155 (14)	−0.0015 (14)
C2	0.0412 (19)	0.043 (2)	0.0399 (17)	0.0012 (15)	0.0208 (15)	−0.0001 (15)
C3	0.0377 (18)	0.041 (2)	0.0366 (17)	−0.0011 (15)	0.0131 (14)	0.0051 (15)
C4	0.0338 (17)	0.0363 (19)	0.0324 (15)	0.0026 (13)	0.0105 (13)	0.0015 (13)

### Geometric parameters (Å, °)

**Table:**

O1—C1	1.365 (4)	C1—C2	1.380 (4)
O1—H1A	0.8200	C1—C4^i^	1.405 (4)
O2—C3	1.305 (3)	C2—C4	1.393 (4)
O2—H2B	0.8200	C2—H2A	0.9300
O3—C3	1.223 (4)	C3—C4	1.480 (4)
O1W—H1WA	0.8485	C4—C1^i^	1.406 (4)
O1W—H1WB	0.8511		
			
C1—O1—H1A	109.5	C4—C2—H2A	119.2
C3—O2—H2B	109.5	O3—C3—O2	123.4 (3)
H1WA—O1W—H1WB	108.2	O3—C3—C4	122.3 (3)
O1—C1—C2	118.3 (3)	O2—C3—C4	114.3 (3)
O1—C1—C4^i^	122.1 (3)	C2—C4—C1^i^	119.0 (3)
C2—C1—C4^i^	119.5 (3)	C2—C4—C3	121.2 (3)
C1—C2—C4	121.5 (3)	C1^i^—C4—C3	119.9 (3)
C1—C2—H2A	119.2		

Symmetry codes: (i) −*x*, −*y*+1, −*z*+2.

### Hydrogen-bond geometry (Å, °)

**Table:**

*D*—H···*A*	*D*—H	H···*A*	*D*···*A*	*D*—H···*A*
O1—H1A···O3^i^	0.82	1.88	2.597 (3)	146
O2—H2B···O1W^ii^	0.82	1.74	2.561 (3)	177
O1W—H1WB···O1^iii^	0.85	1.94	2.786 (3)	175.0
O1W—H1WA···O3^iv^	0.85	2.04	2.809 (3)	150.4

Symmetry codes: (i) −*x*, −*y*+1, −*z*+2; (ii) *x*, *y*, *z*−1; (iii) −*x*+1, −*y*+1, −*z*+2; (iv) *x*, −*y*+3/2, *z*+1/2.
